# Flesh Foods

**Published:** 1900-12

**Authors:** 


**Flesh Foods.** By C. Ainsworth Mitchell. Pp. xv., 336. London: Charles Griffin & Company, Limited. 1900.

Within a remarkably small compass, considering the extent of ground covered, the author presents a very complete conspectus of our knowledge concerning the composition of the meats derived from the numerous animals used for human food. After two introductory chapters dealing with the structure of animal tissues, and such definite chemical bodies as have been found to enter into their constitution, the special subject of this book is introduced by an account of the distinctive features of the flesh of different animals, both domesticated and wild, followed in the succeeding chapter by a description of the methods of examination of meat for soundness and condition, including Eber's tests for putrefaction, and attention is drawn to the practice of producing "blown" meat. A section is here devoted to the digestibility of various meats. Popular ideas on the subject are hardly in accord with the conclusion that beef and veal rank highest in this respect, and are closely followed by salmon; whereas chicken and white fish are attacked by pepsin with much less completeness. In the fifth chapter are briefly but accurately described the methods of fat analysis, in connection with which the author has published useful original work; and then follows a very interesting account of the various methods of preserving meat, a timely note as to the use of nitre in pickling liquor, reminding the reader that the use of this distinctly poisonous salt is hardly questioned, whereas in some quarters no condemnation is too severe for the comparatively innocent borax.

A chapter on sausages, with a detailed account of the methods for the detection of horseflesh, throws considerable light on the composition of these very popular luxuries.

Chapters viii. and xi., dealing with the proteids, the methods employed in their separation and recognition, and the application of these methods to the examination of meat extracts and other preparations, are amongst the most important in the book; and if their perusal leaves the reader with a sensation of uncertainty as to the extent of reliable knowledge in this direction, this is not to be attributed to any remissness on the part of the author, so much as to the fact that this department of practical chemistry is yet in its infancy.

The succeeding chapters, on the influence of cooking, on poisonous flesh, and on the parasites which occur in animal tissues, are not less well conceived than those already described; whilst the final section, dealing with the detection and properties of ptomaines, will be of considerable assistance to the student requiring information.

It will be gathered from the preceding notes that hardly a question can be raised in reference to the subject matter of this volume, to which a reply, more or less complete, will not be found between its covers; and the references which are uniformly appended to notices of processes materially enhance the value of the book.

